# Plant community assembly in suburban vacant lots depends on earthmoving legacy, habitat connectivity, and current mowing frequency

**DOI:** 10.1002/ece3.5985

**Published:** 2020-01-10

**Authors:** Yoichi Tsuzuki, Tomoyo F. Koyanagi, Tadashi Miyashita

**Affiliations:** ^1^ School of Agriculture and Life Sciences The University of Tokyo Tokyo Japan; ^2^ Field Studies Institute for Environmental Education Tokyo Gakugei University Tokyo Japan; ^3^ Present address: Graduate School of Environmental Science Hokkaido University Sapporo Japan

**Keywords:** beta diversity, community disassembly, habitat connectivity, land‐use change, legacy effects, species nestedness, species turnover

## Abstract

In suburban regions, vacant lots potentially offer significant opportunities for biodiversity conservation. Recently, in Japan, due to an economic recession, some previously developed lands have become vacant. Little is known, however, about the legacy of earlier earthmoving, which involves topsoil removal and ground leveling before residential construction, on plant community composition in such vacant lots. To understand (dis)assembly processes in vacant lots, we studied 24 grasslands in a suburban region in Japan: 12 grasslands that had experienced earthmoving and 12 that had not. We surveyed plant community composition and species richness, and clarified compositional turnover (replacement of species) and nestedness (nonrandom species loss) by distance‐based β‐diversities, which were summarized by PCoA analysis. We used piecewise structural equation modeling to examine the effects of soil properties, mowing frequency, past and present habitat connectivities on compositional changes. As a result, past earthmoving, mowing frequency, soil properties, and past habitat connectivity were found to be the drivers of compositional turnover. In particular, we found legacy effects of earthmoving: earthmoving promoted turnover from native grassland species to weeds in arable lands or roadside by altering soil properties. Mowing frequency also promoted the same turnover, implying that extensive rather than intensive mowing can modify the negative legacy effects and maintain grassland species. Decrease in present habitat connectivity marginally enhanced nonrandom loss of native grassland species (nestedness). Present habitat connectivity had a positive effect on species richness, highlighting the important roles of contemporary dispersal. Our study demonstrates that community assembly is a result of multiple processes differing in spatial and temporal scales. We suggest that extensive mowing at local scale, as well as giving a high conservation priority to grasslands with high habitat connectivity at regional scale, is the promising actions to maintain endangered native grassland species in suburban landscapes with negative legacy effects of earthmoving.

## Graphical abstract

Our study investigated the impacts of land‐use changes on grassland plant community in suburban vacant lots. We successfully revealed that multiple factors, including past earthmoving, current mowing, and habitat connectivity, drive multiple compositional shifts (turnover and nestedness) and affect endangered grassland species.

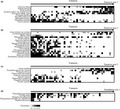

## 1 INTRODUCTION

Habitat loss and degradation caused by human land use in suburban landscapes are a strong driver of biodiversity loss and ecosystem degradation (Deng, Wang, Hong, & Qi, 2009; Foley et al., 2005). Suburban regions, however, have recently drawn attention as areas where conservation activities should be promoted; not only for biodiversity conservation but also for supplying various ecosystem services, such as temperature regulation, air purification, and recreation (Gomez‐Baggethun et al., 2013; Lepczyk et al., 2017). To determine the most effective strategies for biodiversity conservation in suburban landscapes, we need to understand the mechanisms of how human activities alter biological communities in space and time. This then enables managers to prioritize places for conservation and to understand the most effective management actions to achieve conservation objectives.

It is important to note that past land use can be one of the crucial drivers that influence biological communities in suburban regions, that is, legacies of past land use play predominant roles in determining present community composition in human‐disturbed landscapes (Foster et al., 2003; Hermy & Verheyen, 2007). For example, past agricultural activities and settlement have long‐lasting effects on soil physical and chemical properties, and the altered soil affects present plant communities even after the land use ceased (Brudvig, Grman, Habeck, Orrock, & Ledvina, 2013; Dupouey, Dambrine, Laffite, & Moares, 2002; Freschet, Östlund, Kichenin, & Wardle, 2014). Such legacy effects of past anthropogenic activities should be important processes determining present plant communities in suburban landscapes.

Moreover, spatial processes also play a role, as biological communities in suburban landscapes are fragmented by artificial land cover (Liu, He, & Wu, 2016), which affects between‐habitat dispersal maintaining species diversity (Ramalho, Laliberté, Poot, & Hobbs, 2014; Rudolph, Velbert, Schwenzfeier, Kleinebecker, & Klaus, 2017). Spatial dispersal could affect communities with time‐delay in recently fragmented landscapes, which are known as extinction debt or colonization credit (Jackson & Sax, 2010; Kuussaari et al., 2009). For example, contemporary species richness is correlated with past landscape structures, rather than present, indicating the legacy effects of past dispersal (Helm, Hanski, & Pärtel, 2006; Lindborg & Eriksson, 2004). Examining multiple processes differing in their spatial (local to regional) and temporal (past to present) scales is thus necessary to understand community assembly mechanism in suburban regions.

Suburban grassland plant communities in Japan are typically formed by complex spatial and temporal processes. Grasslands in Japan are being fragmented and destroyed due to both land development and abandonment of traditional management, such as mowing and burning (Shoji, Yamamoto, & Suyama, 1995; Yamato & Hattori, 2000). This has led to severe decrease in grassland specialist plant species (Koyanagi & Furukawa, 2013; Yamato, Hattori, & Inagaki, 2001). In suburban regions, earthmoving that involves topsoil removal and ground leveling for residential construction (Sasaki, Morimoto, & Imanishi, 2007; Tamura & Takeuchi, 1980) has caused large declines in grassland area. Currently, as Japan experiences economic recession and a population decline, places previously developed using earthmoving techniques are being abandoned resulting in vacant lots covered with herbaceous plants. These vacant lots offer the potential to boost the conservation of native grassland species that exist in remnant grasslands (Klaus, 2013). It is unknown, however, what legacy effects the earthmoving has on these vacant lots that may serve as potential habitats for grassland species. Added to this is the effect that mowing regimes have on grasslands in suburban landscapes; a management action that has diverse impacts on soil conditions, and consequently grassland biodiversity (Nagata & Ushimaru, 2016).

Earlier studies that examined plant community assembly in human‐disturbed landscapes evaluated both legacy effects and dispersal process (Johnson, Borowy, & Swan, 2018; Turley, Orrock, Ledvina, & Brudvig, 2017). However, the mechanisms of community assembly are not well clarified, because these studies used only alpha (or gamma) diversity as response variables. Focusing on the compositional differences, or beta diversity, between communities in different spatio‐temporal contexts may enable us to infer the processes of community assembly in response to human land‐use alterations. Beta diversity consists of two antithetic (but not exclusive) patterns: species turnover and nestedness (Baselga, 2010). Species turnover reflects the replacement of species from one site to another, which could be the result of environmental sorting, historical processes, or competition (Baselga, 2010; Hill, Heino, Thornhill, Ryves, & Wood, 2017). Nestedness implies that communities with fewer species are the subset of richer communities. Nested assemblages reflect nonrandom species loss, which could be found along the gradient of habitat isolation or environmental harshness (Ulrich, Almeida‐Neto, & Gotelli, 2009). Identifying the mechanisms that drive turnover and nestedness would provide a comprehensive understanding of community assembly in human‐dominated landscapes (Conradi, Temperton, & Kollmann, 2017; Hill et al., 2017).

**Figure 1:**
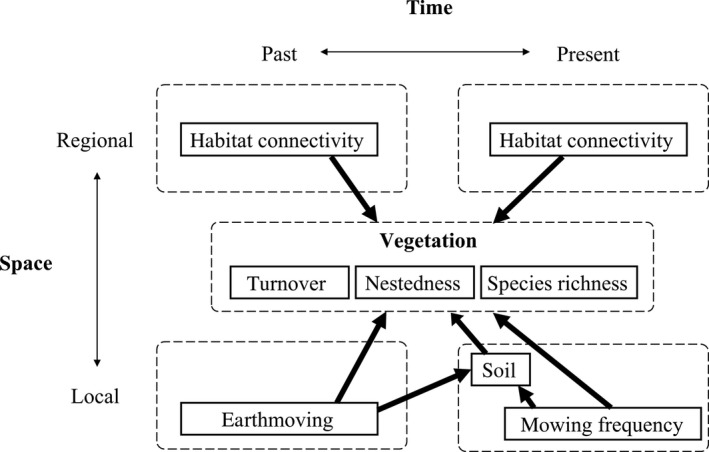
Expected mechanisms of plant community assembly in this research

In this paper, we aimed to identify the mechanisms of community assembly in suburban grassland plant communities in Japan, by relating human activities, soil properties, and landscape structures to species richness, species turnover and nestedness (Figure 1). The specific hypotheses we tested were (a) past earthmoving makes soil oligotrophic by topsoil removal and has long‐lasting legacy effects that promote compositional turnover from native grassland species to species typical for disturbed environments; (b) present mowing increases soil organic matter from plant cut residues (Poeplau, Marstorp, Thored, & Kätterer, 2016) and accelerates nutrient cycling, which subsequently fertilizes soil and promotes species turnover in an opposite way to earthmoving; and (c) habitat connectivity promotes past and present immigration, thereby increasing present species richness and making nested structure (i.e., native grassland species decrease with decreasing habitat connectivity).

## 2 MATERIALS AND METHODS

### 2.1 Study sites

**Figure 2:**
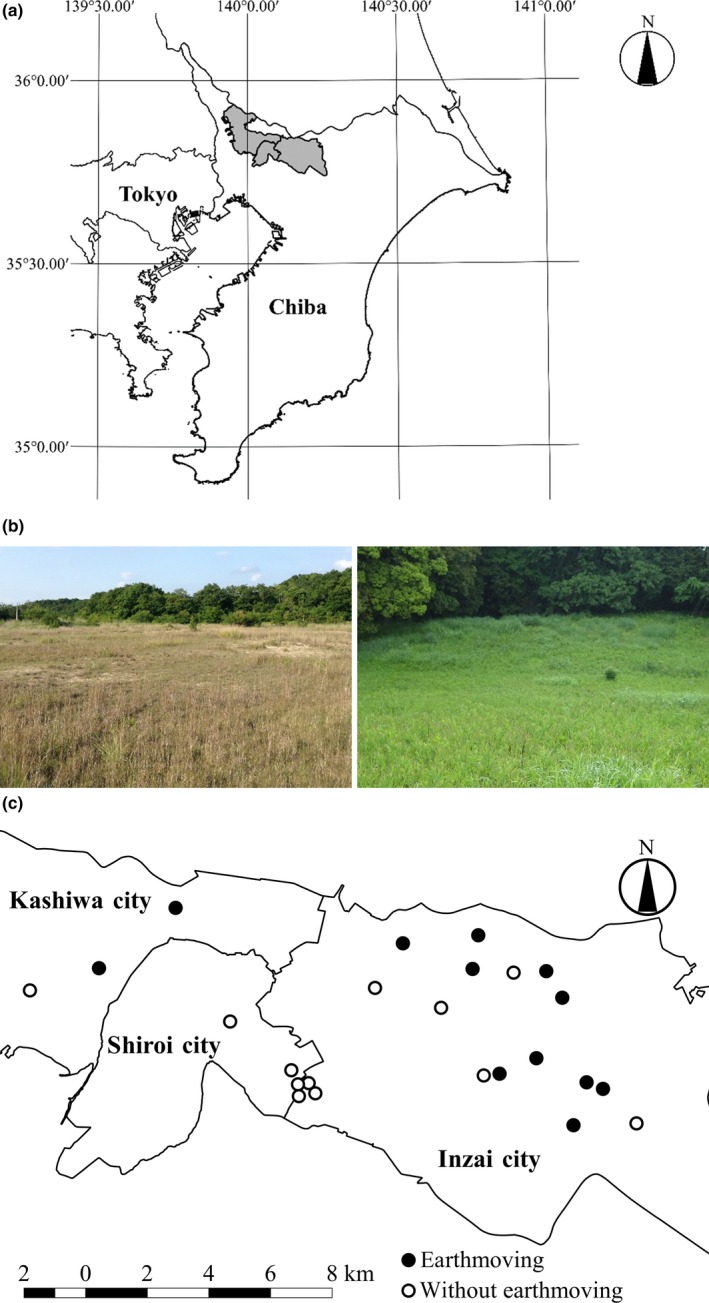
(a) The location of the study region (Shiroi, Inzai, and Kashiwa City in Chiba Prefecture, Japan); (b) a vacant lot that became dominated by herbaceous plants after earthmoving (left) and an old remnant grassland (right); (c) Enlarged view of the study region and the detailed location of 24 study sites. Filled sites have experienced earthmoving, while open sites have not

The study region is located in the northern part of Shimousa Hill in Chiba Prefecture, 30–40 km of northeast of Tokyo metropolitan area, Japan (Figure 2a). Shimousa Hill is one of the largest flat hills in Japan and is covered with layers of volcanic ash on top (Natural History Museum & Institute, Chiba, 2001). Seminatural grasslands are sparsely distributed in this region and can be categorized largely into three types: *Pleioblastus argenteostriatus* and *Miscanthus sinensis*‐dominated grasslands which establish at extensively mowed (once a year or in several years) grasslands, and *Imperata cylindrica*‐dominated grasslands that establish at occasionally mowed (twice a year) grasslands, and *Zoysia japonica*‐dominated grasslands that establish at frequently mowed (more than three times a year) grasslands (Natural History Museum & Institute, Chiba, 2001).

Grasslands in this region had historically been maintained as a pasture for warhorses, but since the middle of 19th century began to be changed to coppice woodlands dominated mainly by pine trees (Miyamoto & Yokohari, 2010). Due to frequent tree cutting, the coppice in these landscapes was generally sparse with short trees, allowing grassland‐like vegetation to persist until the 1960s (Koyanagi, Kusumoto, Yamamoto, & Takeuchi, 2012b; Noda, Kondoh, & Nishihiro, 2019). After this, suburban development took place and earthmoving accompanied by topsoil removal and ground leveling was carried out in this region. Further, as humans became more urbanized, the activity of coppicing declined resulting in a closed canopy with a dark understory in many of the remaining grasslands. More recently, due to economic recession in Japan, some developed lands were underutilized or abandoned and converted to vacant lots dominated by herbaceous plants (Figure 2b). Vegetation in these vacant lots is likely to be influenced by the legacy effects of earthmoving due to building activities. Besides, some places became open grasslands without earthmoving as a result of constant management activities or land‐use changes other than building construction (e.g., cultivation).

Since the study region contains fragmented grasslands with different land‐use histories as described above, this system was ideal for examining the multiple assembly processes including anthropogenic effects. We selected 24 grasslands based on the presence/absence of earthmoving (earthmoving: 12 grasslands, no earthmoving: 12 grasslands, Figure 2c and Table S1).

### 2.2 Vegetation and mowing frequency

Vegetation surveys were carried out in May, July, and September of 2017. In May, each grassland was divided into one to four sections based on the difference in dominant herbaceous species (see Table S1 for the number of transects in each grassland). One 9‐m transect was established in each section, and five 1 m × 1 m quadrats were placed along the transect at 1‐m intervals. These transects and quadrats were fixed and were also examined in later surveys (July, September). In each survey, all species present in each quadrat were recorded. Presence/absence at a given quadrat was determined based on whether or not it was present at least once in three surveys. Occurrence of a given species per transect was then calculated as the number of quadrats in which that species was present. Mowing frequency was estimated for each transect by asking landowners and managers or, failing that, the signs of having been mown at the time of field surveys.

### 2.3 Soil analysis

**Figure 3:**
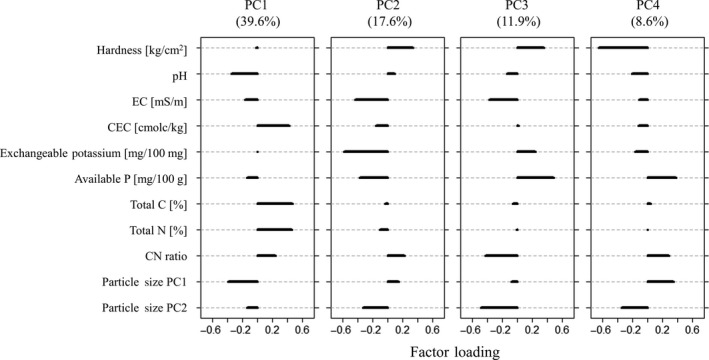
Factor loadings of 4 primary components (PC1 to PC4) of soil properties. 77.8% of the total variance was explained. Horizontal axis shows the value of factor loadings

Soil samples were collected in June 2017. Before sampling, soil hardness was measured at the center of the quadrats of the vegetation survey with a Yamanaka‐type soil hardness tester (Fujiwara Scientific Co., Japan), and the mean hardness of 5 quadrats was calculated for each transect. Soil cores (5 cm diameter, 5 cm height) were sampled from the topsoil at the center of the quadrats, and the mixed soils were used for analysis. After air‐drying for 5 days, samples were ground with a mortar and pestle, and sifted with a 2‐mm sieve. The following 9 chemical and physical properties were measured, that is, pH, EC (electrical conductivity), CEC (cation exchange capacity), exchangeable potassium, available phosphorus, total C, total N, CN ratio, and particle size composition (for detailed methods see Table S2). Principal component analysis (PCA) was carried out to summarize particle size composition into 2 axes representing predominance of medium particles and particle size, respectively (Figure S1). Using these two principal components and the other eight soil properties, an additional PCA was conducted to produce variables that represented integrated soil properties. Physical and chemical features of the resultant primary components were identified by factor loadings (Figure 3) The first four axes, which accounted for 77.8% of the total variance, were used for the subsequent analyses.

### 2.4 Land‐use survey

Aerial photographs and satellite images of the study region taken in 20 different time points in the last 70 years were used for the reconstruction of land‐use history (i.e., 1947, 1949, 1966, 1969, 1975, 1979, 1984, 1989, 1992, 1995, 1996, 1997, 1998, 2001, 2006, 2008 [Geospatial Information Authority of Japan (GSI), http://mapps.gsi.go.jp/maplibSearch.do#1], 2009, 2012, 2014, and 2017 [Google Earth]). According to the vegetation survey in 1968 (just before the town development), pine‐dominated forests were occupied by grassland‐like vegetation harboring grassland species. Therefore, all forests were regarded as grasslands until 1970, which is the period before region‐wide earthmoving. Forests after 1970 were regarded as genuine forests because coppicing management was abandoned, resulting in dark understory with no grassland vegetation. We then identified the presence/absence of earthmoving, and the year when a focal study site became a grassland (from arable land, building, and forest). The occurrence of earthmoving was judged by the trace of topsoil removal or the presence of heavy equipment for earthmoving. The initial year (Table S1) was estimated as the mid‐point of years before and after a given land‐use change was identified by photographs. However, when ongoing earthmoving (such as partially modified land use with heavy equipment) was recognized in study sites that subsequently became grasslands after earthmoving, the year when the image was taken was regarded as the initial year. In old grasslands that had no history of land conversion during the past 70 years, the initial year was set to 1947, when the oldest aerial photograph was taken.

### 2.5 Habitat connectivity

The area of the surveyed grasslands and connectivity to surrounding grasslands were measured for the following four periods (each approximately 18 years) in the past 70 years: (a) before 1966 (no major landscape change occurred), (b) from 1967 to 1984 (extensive new‐town development and earthmoving began), (c) from 1985 to 2001 (land development progressed further), (d) from 2002 to 2017 (land‐use change gradually decreased). Aerial photographs taken in 1966, 1984, and 2001 (acquired from GSI website) and ESRI satellite image taken in 2017, covering the whole study region, were used. We defined initial and present landscapes for each grassland as those in the period including the initial year and period (d), respectively.

We extracted grasslands that had experienced no earthmoving within a 500 m radius buffer from study sites. Habitat connectivity was then measured for each study site as the area proportion of the extracted grasslands to the buffer. The 500m buffer size was determined based on earlier studies that examined the distribution of grassland species or of species diversity in suburban landscapes (Duguay, Eigenbrod, & Fahrig, 2007; du Toit, Kotze, & Cilliers, 2016; Koyanagi, Akasaka, Oguma, & Ise, 2017; Vakhlamova, Rusterholz, Kanibolotskava, & Baur, 2014).

All spatial analyses were conducted on QGIS 2.8.18 (QGIS Development Team, 2018). Aerial photographs for the first three periods were georeferenced, and the ESRI satellite image for the fourth period was acquired via QuickMapServices plugin.

### 2.6 Species richness and ordination

**Figure 4:**
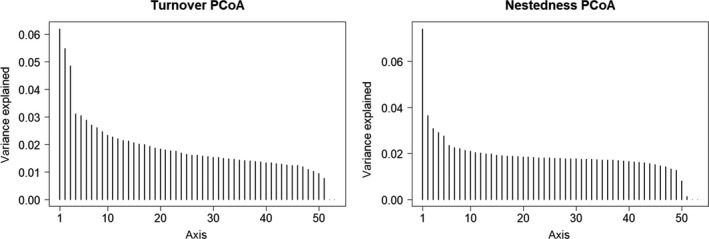
Explained variance of PCoA axes. In turnover PCoA, axis 4 or later had low explanatory power compared to the first three axes (axis 1:6.2%; axis 2:5.5%; axis 3:4.9%). In nested PCoA, only the first axis had prominent explanatory power (7.4%)

Species richness was calculated per transect as a measure of alpha diversity. For all transect pairs, beta diversity was first calculated from Sorensen dissimilarity ($\beta_{\mathrm{sor}}$) and then decomposed into species turnover (replacement of species) and nestedness (nonrandom species loss; Baselga, 2010),

(1) $$\beta_{\mathrm{sor}}=\beta_{\mathrm{sim}}+\beta_{\mathrm{nes}}$$

where $\beta_{\mathrm{sim}}$ is Simpson's dissimilarity index arising from species turnover, while $\beta_{\mathrm{nes}}$ represents nestedness. These β diversities were computed by an R package betapart (Baselga & Orme, 2012). To evaluate compositional changes underlying species turnover and nestedness, principal coordinate analysis (PCoA) was performed separately for $\beta_{\mathrm{sim}}$ and $\beta_{\mathrm{nes}}$. Based on the explained variance of each PCoA axis, 3 axes from $\beta_{\mathrm{sim}}$ and one axis from $\beta_{\mathrm{nes}}$ were used for the following analysis, as they had much higher explanatory power in comparison to the rest of the axes (Figure 4).

To identify characteristic species for ordination axes, Pearson's correlation coefficients between PCoA axes and species occurrence of all 247 species recorded were calculated. Statistical significance of the correlation coefficients was tested by a randomization test with 9,999 replications, and the significance threshold of 0.05 was adjusted with the sequential Bonferroni method. For species having a significant correlation with any of the four axes, species characteristics (height, longevity, origin, and grassland species or not) were examined based on earlier studies (Kaneko, Mimura, Amano, & Hasegawa, 2009; Koyanagi & Furukawa, 2013; Miyawaki, 1986; Natural History Museum & Institute, Chiba, 2003) to understand plant compositional shifts along each ordination axis. Correlations among the four ordination axes and species richness were also tested using sequential Bonferroni method.

### 2.7 Statistical analysis

We used piecewise structural equation model (piecewise *SEM*) to estimate causal effects in a priori hypotheses (Figure 1). As with traditional *SEM*, piecewise *SEM* can quantify causal networks but it differs in its estimation procedures: the whole causal network is decomposed into a series of regression models, each of which is evaluated separately with a generalized linear model (Lefcheck, 2016; Shipley, 2009). Thus, piecewise *SEM* can operate with limited sample sizes and incorporate various model features, such as random effects and hierarchical structure.

The regression model we used here is a hierarchical linear model (Raudenbush & Bryk, ), because our study has two data hierarchies: transect‐level (lower level) and site‐level (upper level). The model for transect *i* in site *j* is as follows:

(2) $$Y_{\mathrm{ij}}=b_{0j}+b_{1}W_{1}+b_{2}W_{2}+\cdots +e_{\mathrm{ij}}$$

(3) $$b_{0j}=c_{0}+c_{1}X_{1}+c_{2}X_{2}+\cdots +u_{j}$$

The transect‐level data were used for Equation (2), and the site‐level data were used for Equation (3). *Y_ij_* is a transect‐level response variable, while *W* and *X* are, respectively, the transect‐level and site‐level explanatory variables. *b*_oj_ is the intercept for study site *j* and is modeled with site‐level variables. *e*_ij_ is the error term, and *u_j_* is a random effect of study site. We established five models, each of which used one of the four ordination axes of β diversities or species richness as a response variable. The explanatory variables in Equation (2) (W) were four soil PCs and mowing frequency, and those in Equation (3) (*X*) were initial and present connectivity and earthmoving. We also evaluated causal effects from mowing frequency or earthmoving to soil properties (four soil PCs), using mowing frequency as *W* in Equation (2) and earthmoving as *X* in Equation (3). Analyses were conducted in R 3.5.2 (R Development Core Team, 2018). Function “lme” in package “nlme” was used for each hierarchical linear model, and function “psem” in package “piecewiseSEM” was used for specifying the whole causal network (Lefcheck, 2016). All response and explanatory variables were standardized.

Our analyses paid attention to the following statistical problems, (a) correlations among unlinked factors and (b) spatial autocorrelation among study sites.

In piecewise *SEM*, the goodness‐of‐fit of the whole causal network is evaluated by directed separation, which tests (conditional) independence for the basis set, that is, pairs of variables that are not linked directly in Figure 1 (e.g., earthmoving‐mowing pair), using Fisher's *C* (Lefcheck, 2016; Shipley, 2009). It should be noted that some pairs in the basis set are (conditionally) interrelated because of plausible causalities that are of no interest. One such relationship is a temporal correlation between initial and present habitat connectivity (correlation coefficient = 0.57, *p* = .004). Moreover, as earthmoving was carried out in a spatially continuous manner in the study region, negative correlations between earthmoving and habitat connectivity were observed (earthmoving and initial connectivity: −0.66, *p* < .001; earthmoving and present connectivity: −0.40, *p* = .053). As these associations could severely decrease the goodness‐of‐fit of the model due to collinearity, they were excluded from the basis set and treated as correlated errors.

To account for spatial autocorrelation, we first defined neighboring relationships using Delaunay Triangulation weighted by distance and then conducted Moran's I test (*α* = .05) for the site‐level value of each variable. As a result, turnover axis 3, soil PC1, earthmoving, and present habitat connectivity had significant spatial autocorrelations (Table S3). When assessing the causal effects among these variables, we incorporated 6 additional variables that are significant in MEM (Moran's eigenvector maps; Dray, 2018) as covariates.

## 3 RESULTS

### 3.1 Community compositions represented by PCoA axes

**Figure 5:**
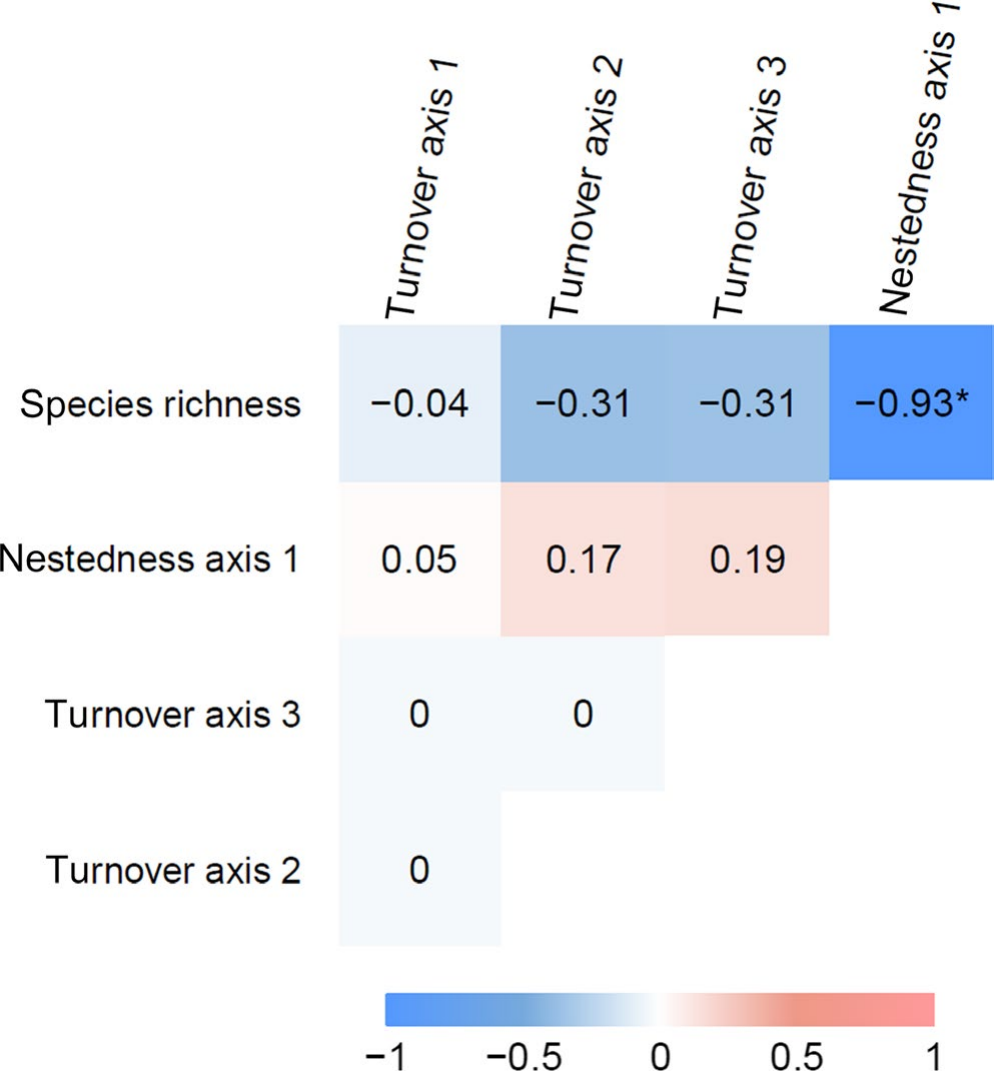
Correlation among 4 ordination axes and species richness. Correlation coefficients are shown with a color gradient from blue (correlation coefficient = −1) to red (correlation coefficient = 1). * denotes significant correlation (*p* < .05, adjusted by sequential Bonferroni method)

**Figure 6:**
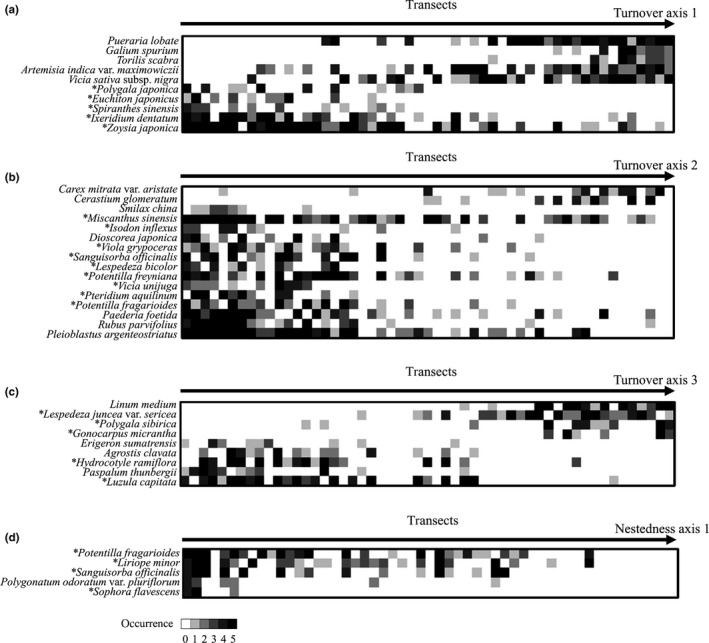
Species composition patterns represented by the 4 PCoA axes: turnover axis 1 (a), axis 2 (b), axis 3 (c), and nestedness axis 1 (d). For each axis, occurrences of species that had significant correlation (see Table S4) are presented on a transect‐level. Transects are ordered from left to right in an ascending order of the PCoA axis. * denotes grassland species

Four PCoA axes of beta diversity were independent each other (Figure 5) and reflected different aspects of community composition (Figure 6, Table S4). Turnover axis 1 represents a gradient from native grassland species with low height (negatively correlated) to tall herbaceous species and vines characteristic to roadside or abandoned crop fields (positively correlated). Turnover axis 2 represents a gradient from native grassland species (negatively correlated) to weeds in arable lands or roadside (positively correlated). Species correlated with turnover axis 3, either positively or negatively, were typical for disturbed environments (e.g., roadside and vacant lots), indicating that turnover axis 3 represents species replacement among these species. Nestedness axis 1 correlated negatively with native grassland species, representing gradual loss of grassland species with increasing value of nestedness axis 1. This gradual loss might underlie decrease in species richness, since nestedness axis 1 was significantly correlated with species richness (Figure 5). It should be noted that species identities of native grassland species were different between axes, although they partly overlapped (Figure 6, Table S4), indicating that different subsets of grassland species are represented in different ordination axes.

### 3.2 Causal effects of earthmoving, mowing, soil properties and habitat connectivity on community composition and species richness

**Figure 7:**
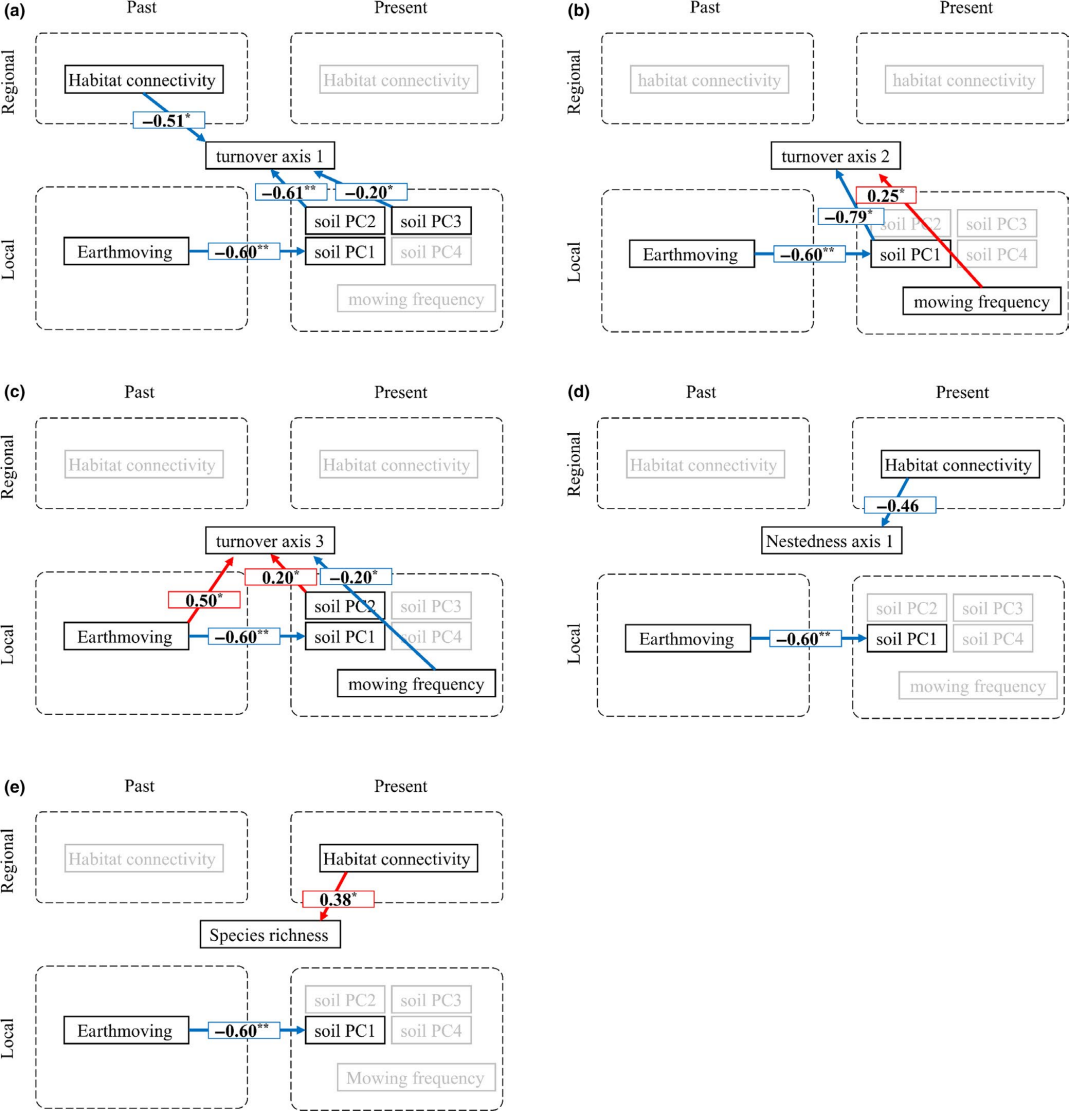
Results of piecewise structural equation model: (a) turnover axis 1, (b) turnover axis 2, (c) turnover axis 3, (d) nestedness axis 1, and (e) species richness. Only significant (**p < .01; *p < .05) paths are shown for convenient visualization, except marginally significant (*p* < .1) paths in (d) nestedness axis 1. Red and blue arrows indicate positive and negative effects, respectively. Factors without significant paths coming in or out of are shown in semitransparent. Fisher's *C* of all the 5 diagrams satisfied *p* > .05, indicating enough goodness‐of‐fit

Turnover axis 1 was affected by soil PC2 (negatively characterized by EC, exchangeable K, and available P), soil PC3 (positively characterized by hardness and available P, and negatively by EC, CN ratio, and particle PC 2), and initial habitat connectivity. All of these variables had significant negative direct effects on the turnover axis 1 (Figure 7a), indicating that they promoted compositional turnover from tall herbaceous species and vines to native grassland species.

There was a negative direct effect of soil PC1 and a positive direct effect of mowing frequency on turnover axis 2 (Figure 7b). In other words, high soil PC1 (low pH and high CEC, total C, total N, and particle PC1) or low mowing frequency promoted the compositional turnover from weeds in arable lands or roadsides to native grassland species. Earthmoving negatively affected soil PC1 and thereby indirectly increased the score of turnover axis 2, which was in parallel with the decrease of grassland species.

There was a positive direct effect of past earthmoving and a negative direct effect of mowing frequency on turnover axis 3 (Figure 7c). This suggests that the past and present anthropogenic activities induce compositional turnover of plant species characteristic to disturbed environments in opposite directions. Moreover, soil PC2 (negatively characterized by EC, exchangeable K, and available P) also had a positive direct effect, inducing species turnover in the same direction as earthmoving.

There were no variables that significantly affected nestedness axis 1, but there was a marginal negative direct effect from present habitat connectivity (Figure 7d). This suggests that decrease in present connectivity may enhance nonrandom loss of native grassland species, since nestedness axis 1 correlated negatively with the occurrence of native grassland species.

There was a direct positive effect of present habitat connectivity on species richness (Figure 7e), implying that present immigration from surrounding grasslands supports high species richness at the focal habitat. Fisher's *C* of all the five causal networks presented in Figure 7 satisfied *p* > .05 in directed separation, indicating sufficient goodness‐of‐fit.

## 4 DISCUSSION

We have shown that four ordination axes were subjected to different explanatory variables. This could be attributed to interspecific variations in crucial factors for presence/absence, as the four ordination axes represent different compositional changes. We will discuss assembly mechanisms underlying these compositional shifts in detail below.

### 4.1 Legacy effect of earthmoving and mowing frequency

Our results indicate that past earthmoving altered soil properties, and that the altered soil triggered turnover from native grassland species to weeds inhabiting arable lands or roadsides (Figure 7b), supporting our first hypothesis. In contrast to our second hypothesis, this turnover was not mediated by mowing frequency; mowing frequency also induced the same direction of species turnover, decreasing native grassland species (Figure 7b).

Past land use is known to cause long‐lasting changes in soil properties, which in turn affects plant communities (Brudvig et al., 2013; Dupouey et al., 2002; Freschet et al., 2014; Isbell, Tilman, Polasky, Binder, & Hawthorne, 2013; Mattingly & Orrock, 2013). However, few researches have investigated the legacy effects of earthmoving, despite this practice being conducted at an increasing rate worldwide as a result of global human population growth (Hooke, 2000). We have shown that past earthmoving increased pH, decreased CEC, total C, total N, and medium particles, and that native grassland species are negatively affected by the edaphic changes induced by earthmoving. Increase in pH and decrease in CEC, total C, and total N caused by earthmoving can be attributed to topsoil removal, since acidic soil organic matters like humus originate mainly from topsoil. The predominance of medium particles might be due to the loss of large and small particles, resulting from breakup of large particles caused by earthmoving and the removal of small particles such as clay. It appears that environmental filtering plays a role in subsequent processes: the altered soil conditions are unsuitable for native grassland species, thus reducing their occurrence. Soil in the study region is mostly acidic, as a result of the weathering of volcanic ash. The soil is also rich in organic matter, but plant‐available phosphate is poor which is caused by high phosphate absorption coefficient (Hiradate, Morita, & Kusumoto, 2008; Shoji, Hayashi, Kohyama, & Sasaki, 2010). While native grassland species are very common in such soil environments, they occur less in high pH soil environments disturbed by land development or fertilization, where alien species dominate instead (Hiradate et al., 2008). This finding agrees with our result showing indirect effects of earthmoving on soil and vegetation.

Our finding that mowing frequency had a negative effect on native grassland species might seem inconsistent with earlier studies showing that mowing maintains grassland environment and contributes to the establishment of grassland species in general (Brys, Jacquemyn, Endels, de Blust, & Hermy, 2004; Cousins & Eriksson, 2002; Ehrlén, Syrjänen, Leimu, Garcia, & Lehtilä, 2005; MacDougall & Turkington, 2007; Valkó, Török, Matus, & Tóthmérész, 2012). However, frequent mowing observed in our study sites resulted in very low plant height, indicating intensive rather than moderate mowing intensity was implemented in these grasslands. In fact, some of the grasslands in our study region received high frequency, excessive mowing (more than four times a year), which inhibited the establishment of grassland species (Kaneko, Tanikawa, & Hasegawa, 2012). Thus, a wide range of mowing gradient, including intensive mowing, may explain the observed negative impacts of mowing on grassland species occurrence.

Earthmoving and mowing frequency affected differently the compositional turnover among species typical in disturbed environments (Figure 7c). The species turnover was also correlated with available P, EC, and exchangeable K, all of which can be interpreted as soil nutrient components. These indicate that species positively correlated with turnover 3 tend to occur in oligotrophic environment while those correlated negatively in fertile condition. Therefore, this compositional turnover may reflect species sorting by nutritional gradients arising from interspecific variation in the tolerance to oligotrophic environment. In Europe, species habitat requirements are quantified by Ellenberg indicator values (Diekmann, 2003), but there are no counterpart proxies in Japan, making it difficult to confirm the possible effects of the nutritional gradient. Evaluation of species environmental requirements is needed to fully understand the mechanisms of compositional changes.

### 4.2 Landscape structure and its consequences on species composition

Habitat connectivity had significant effects on both species richness and turnover (Figure 7a,e), as well as marginally significant effect on nestedness (Figure 7d), in a way that boosts species richness or the occurrence of native grassland species. Therefore, our third hypothesis that habitat connectivity increases species richness and native grassland species was supported, implying dispersal limitation on species richness and the occurrence of grassland species in fragmented grasslands. Species richness and nestedness (although marginally) were affected by present connectivity, but not by past connectivity (Figure 7d,e), indicating that current immigration from surrounding habitats maintains high species richness and grassland specialist plants. In contrast to this result, time‐delayed responses to landscape change (Jackson & Sax, 2010) have been reported for species richness (Bagaria, Helm, Roda, & Pino, 2015; Helm et al., 2006; Koyanagi, Kusumoto, Yamamoto, Okubo, et al., 2012a; Lindborg & Eriksson, 2004; Otsu, Iijima, Nagaike, Takuo, & Hoshino, 2017) and nested components (Koyanagi, Furukawa, & Osawa, 2018) of grassland plant communities. Cousins (2009) found that time‐delayed response is detected in regions where contemporary landscapes retain >10% of traditional habitats. As our study region maintains only 2.8% of remnant grasslands of those in 1880s (Noda et al., 2019), delayed responses may not have been observed.

There might be time‐delayed response, however, in the turnover pattern since initial, rather than present, habitat connectivity had a significant effect (Figure 7a). This result highlights the importance to evaluate β diversity when considering community dynamics at landscape scales, because evaluation of species richness (or alpha diversity) alone did not reveal the impact of the past landscape structure. The significant effect of initial connectivity indicates that grassland species had immigrated more to grasslands with higher initial connectivity and persisted thereafter, resulting in high occurrence in the present community (Jackson & Sax, 2010). In contrast, grasslands with low connectivity initially might have suffered from dispersal limitation in early successional stage, and instead, species associated with roadside or abandoned crop fields immigrated from the surrounding human‐dominated landscape.

### 4.3 Suggestions for the conservation of grassland vegetation

We have successfully extracted four ordination axes of compositional turnover and nestedness, which were shaped by different assembly processes. Hence, the present community is a complex composite of multiple compositional shifts. Our study is notable for estimating the mechanisms of each assembly pattern with reference to various processes, including legacy effects of earthmoving, spatial dispersal, and time‐delayed response to landscape changes.

These findings provide implications for the conservation of grassland vegetation. Firstly, the results we show on the legacy effects of earthmoving and mowing frequency would be helpful for local management. Earthmoving had a negative legacy effect on grassland species. Considering that mowing frequency had a significant negative effect on the same species, improving mowing regimes in ways such as lowering frequency and intensity could possibly buffer against the worst of the legacy effects of earthmoving. In the study region, the time elapsed since the practice of mowing started plays an important role in the survival of endangered grassland plants (Kaneko, Akeboshi, Hasegawa, & Miyashita, 2013). Quantifying the causal effects of time, therefore, would be needed to suggest more effective management strategies.

Secondly, the effect of habitat connectivity revealed by our study is helpful for prioritizing grassland habitats for conservation at a regional scale. We found that initial and present habitat connectivity is, in some way, a determinant of grassland specialist occurrence. Therefore, in addition to the grasslands where many endangered species currently persist, it is desirable to prioritize places which are surrounded by grasslands that do not have a history of earthmoving. This would make the best use of immigration from surrounding grasslands and support grassland specialist occurrence.

As we have demonstrated, communities are made up of multiple compositional subsets which are shaped by different assembly processes. If we had focused only on species richness, we would have missed important anthropogenic impacts including legacy effects and would not have suggested conservation implications outlined above. Therefore, our approach of decomposing community composition into multiple assembly patterns (i.e., turnover and nestedness) and evaluating anthropogenic impacts on each of them is necessary for the study of biodiversity conservation.

## CONFLICT OF INTEREST

None declared.

## AUTHOR CONTRIBUTIONS

Y. Tsuzuki, T. F. Koyanagi, and T. Miyashita conceived the ideas and designed the methodology; Y. Tsuzuki collected data and performed the statistical analyses; Y. Tsuzuki, T. F. Koyanagi, and T. Miyashita discussed the results; Y. Tsuzuki led the writing of the manuscript. All authors contributed critically to the drafts and gave final approval for publication.

## Data Availability

Data on species occurrence, soil properties, past and present habitat connectivity, land‐use changes, and mowing frequency are all available from the Dryad Digital Repository (https://doi.org/10.5061/dryad.kh1893226).
