# Supplementary material for: Plant community assembly in suburban vacant lots depends on earthmoving legacy, habitat connectivity, and current mowing frequency
Source: Ecol Evol. 2020 Jan 10;10(3):1311–23. doi: 10.1002/ece3.5985 (PMC7029082; doi:10.1002/ece3.5985)
Supplement: Supplementary file 1 [file ECE3-10-1311-s001.docx]

**Table S1** Temporal change in site area, the initial year, presence/absence of earthmoving, and the number of transects established in each study site. Area of some sites were 0 in early periods because they had been utilized as other land use, such as arable lands and buildings, before becoming grasslands. Since there were not enough replicates for each past land use to validate its effect, we focused only on the presence/absence of earthmoving.

| site area [ha] | | | | the initial year | presence/absence of earthmoving | No. of transects |
| --- | --- | --- | --- | --- | --- | --- |
| period (a)  (1966) | period (b)  (1984) | period (c)  (2001) | period (d)  (2017) |  |  |  |
| 1.499 | 1.806 | 1.781 | 1.306 | 1947 | 0 | 4 |
| 0.972 | 1.074 | 1.132 | 0.888 | 1947 | 0 | 2 |
| 1.261 | 0.669 | 1.478 | 0.833 | 1947 | 0 | 2 |
| 0.76 | 1.502 | 1.09 | 0.707 | 1947 | 0 | 4 |
| 0.265 | 1.25 | 0.718 | 0.498 | 1947 | 0 | 3 |
| 1.599 | 0.513 | 0.812 | 0.465 | 1947 | 0 | 3 |
| 1.362 | 1.374 | 2.335 | 0.414 | 1947 | 0 | 1 |
| 0 | 0.327 | 1.327 | 1.206 | 1970.5 | 0 | 2 |
| 0 | 0.728 | 1.076 | 0.281 | 1970.5 | 1 | 1 |
| 0 | 0.761 | 0.952 | 1.134 | 1977 | 0 | 1 |
| 0 | 0.72 | 0.57 | 0.735 | 1977 | 0 | 3 |
| 0 | 2.189 | 0.086 | 0.081 | 1977 | 1 | 2 |
| 0 | 0.364 | 0.674 | 2.255 | 1979 | 1 | 3 |
| 0 | 1.316 | 1.038 | 0.982 | 1981.5 | 0 | 2 |
| 0 | 0.183 | 0.255 | 0.247 | 1981.5 | 1 | 2 |
| 0 | 3.644 | 0.204 | 0.057 | 1981.5 | 1 | 1 |
| 0 | 0 | 0.039 | 0.029 | 1986.5 | 0 | 1 |
| 0 | 0 | 0.936 | 0.843 | 1992 | 1 | 2 |
| 0 | 0 | 2.699 | 1.214 | 1994 | 1 | 3 |
| 0 | 0 | 0.467 | 0.129 | 1994 | 1 | 2 |
| 0 | 0 | 0.349 | 0.127 | 1996.5 | 1 | 2 |
| 0 | 0 | 1.059 | 0.499 | 1998 | 1 | 3 |
| 0 | 0 | 0.792 | 0.173 | 1998.5 | 1 | 2 |
| 0 | 0 | 0 | 0.666 | 2010.5 | 1 | 2 |

**Table S2** The methods for measuring the nine soil properties. Measured values of CEC, exchangeable potassium, and available phosphate were adjusted to the amount per 1g absolute dry soil, by multiplying the weight ratio of absolute dry soil to air-dried soil. The weight ratio was estimated by the weight of air-dried soil after drying (110℃, 15 hours) divided by the weight before drying.

|  | methods |
| --- | --- |
| pH (H_2_O) | Dip the samples in purified water for 24 hours, and measure the pH of the solution with pH meter |
| EC | Dip the samples in purified water, shake them for 24 hours (100rpm, 30℃), and measure the EC of the solution with EC meter |
| CEC | Peech method (substitute cations to NH_4_^+^, extract NH_4_^+^, and measure the concentration by neutralization titration with H_2_SO_4_aq) |
| exchangeable potassium | Extract with 1M NH_4_CH_3_COOaq, and measure the concentration by atomic absorption spectrometry |
| available phosphate | Extract by Bray-2 method, color the extracted phosphate by molybdenum blue method, and measure the concentration by spectrophotometry |
| total C | Pregl-Dumas method |
| total N | Pregl-Dumas method |
| CN ratio | Calculate the ratio [(total C)/(total N)] |
| particle composition | Classify each sample into 6 size classes (-53µm, 53-106µm, 106-250µm, 250-500µm, 0.5-1mm, 1-2mm) with 5 sieves (1mm, 500µm, 250µm, 106µm, 53µm), measure the weight ratio for each size class, and then carry out principal component analysis to reduce the dimension from 6 to 2 |

**Table S3** Spatial autocorrelation of each variable was validated by Moran’s *I* test. Those variables with * were significantly spatially autocorrelated (*P* < 0.05).

|  | **Moran’s *I*** | ***P* value** |
| --- | --- | --- |
| turnover axis 1 | -0.119 | 0.7464 |
| turnover axis 2 | 0.030 | 0.2608 |
| **turnover axis 3** | **0.248** | **0.0057*** |
| nestedness axis 1 | 0.082 | 0.1539 |
| species richness | 0.082 | 0.1327 |
| **earthmoving** | **0.293** | **0.0020*** |
| mowing frequency | 0.001 | 0.3302 |
| **soil PC1** | **0.385** | **0.0001*** |
| soil PC2 | -0.090 | 0.6585 |
| soil PC3 | 0.080 | 0.1030 |
| soil PC4 | -0.002 | 0.3541 |
| **initial habitat connectivity** | **0.253** | **0.0044*** |
| present habitat connectivity | 0.114 | 0.0832 |

**Figure S1** Factor loadings of the two principal components (PCs) of particle composition. As for PC1, because only factor loadings of medium classes (0.106~0.25mm and 0.25～0.5mm) are positive, PC1 is likely to represent the predominance of medium particles. There is a clear correspondence between factor loadings of PC2 and particle size: large classes (1~2mm, 0.5~1mm, 0.25~0.5mm) have positive values and small classes (0.106~0.25mm, 0.053~0.106mm, ~0.053mm) have negative values, indicating that PC2 represents particle size.


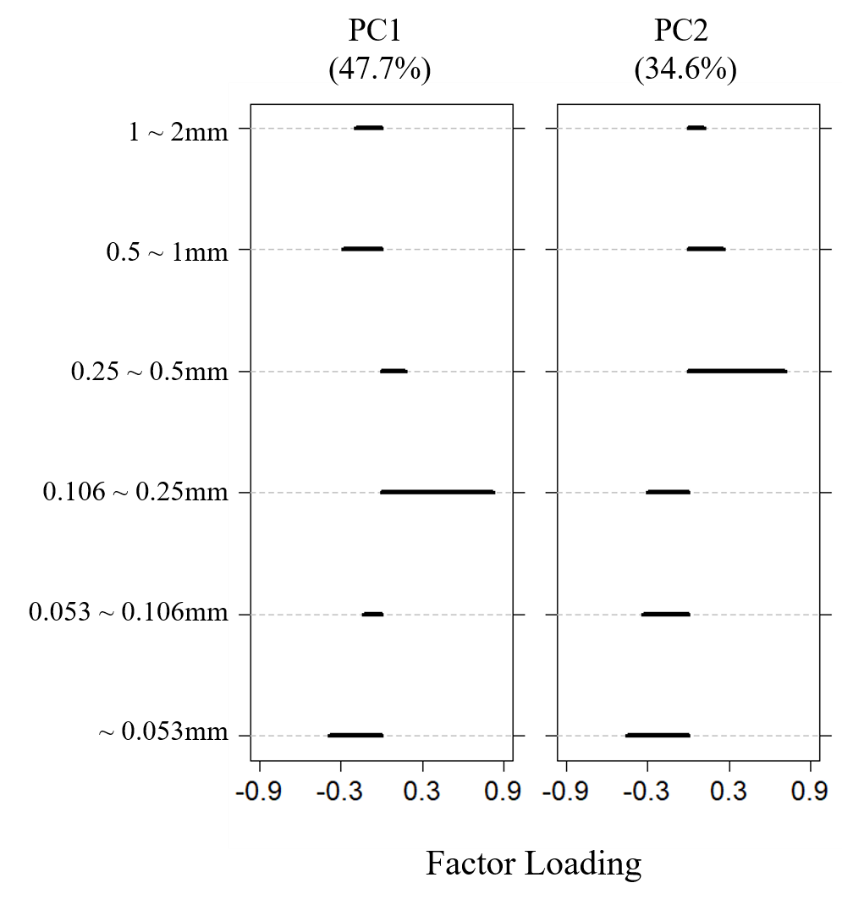


**Table S4** Species whose occurrence were significantly correlated with each ordination axis. Nomenclature is based on The Plant List v 1.1 (www.theplantlist.org).

| **Species Name** | **r** | **height** | **longevity** | **origin** | **grassland species** |
| --- | --- | --- | --- | --- | --- |
|  |  |  |  |  |  |
| **Species turnover axis 1** |  |  |  |  |  |
| *Pueraria lobata* | 0.723 | vine | p | native |  |
| *Galium spurium* | 0.602 | medium | a | native |  |
| *Torilis scabra* | 0.579 | medium | a | native |  |
| *Artemisia indica* var. *maximowiczii* | 0.543 | high | p | native |  |
| *Vicia sativa* subsp. *nigra* | 0.539 | vine | a | native |  |
| *Polygala sibirica* | -0.431 | low | p | native | ○ |
| *Euchiton japonicus* | -0.453 | low | p | native | ○ |
| *Spiranthes sinensis* | -0.526 | low | p | native | ○ |
| *Ixeridium* *dentatum* | -0.626 | low | p | native | ○ |
| *Zoysia japonica* | -0.626 | low | p | native | ○ |
|  |  |  |  |  |  |
| **Species turnover axis 2** |  |  |  |  |  |
| *Carex* *mitrata* var. *aristata* | 0.455 | medium | p | native |  |
| *Cerastium glomeratum* | 0.416 | low | b | alien |  |
| *Smilax china* | -0.484 | shrub | p | native |  |
| *Miscanthus sinensis* | -0.496 | high | p | native | ○ |
| *Isodon inflexus* | -0.521 | medium | p | native | ○ |
| *Dioscorea japonica* | -0.522 | vine | p | native |  |
| *Viola grypoceras* | -0.524 | low | p | native | ○ |
| *Sanguisorba officinalis* | -0.547 | high | p | native | ○ |
| *Lespedeza bicolor* | -0.570 | shrub | p | native | ○ |
| *Potentilla freyniana* | -0.575 | low | p | native | ○ |
| *Vicia unijuga* | -0.592 | medium | p | native | ○ |
| *Pteridium aquilinum* | -0.594 | medium | p | native | ○ |
| *Potentilla fragarioides* | -0.616 | low | p | native | ○ |
| *Paederia foetida* | -0.624 | vine | p | native |  |
| *Rubus parvifolius* | -0.710 | shrub | p | native |  |
| *Pleioblastus argenteostriatus* | -0.732 | shrub | p | native |  |
|  |  |  |  |  |  |
| **Species turnover axis 3** |  |  |  |  |  |
| *Linum medium* | 0.689 | medium | a | alien |  |
| *Lespedeza juncea* var. *sericea* | 0.660 | medium | p | native | ○ |
| [*Polygala sibirica*](http://ylist.info/ylist_detail_display.php?pass=5339) | 0.495 | low | p | native | ○ |
| *Gonocarpus micranthus* | 0.444 | low | p | native | ○ |
| *Erigeron sumatrensis* | -0.418 | high | b | alien |  |
| *Agrostis* *clavata* | -0.449 | medium | p | native |  |
| *Hydrocotyle ramiflora* | -0.486 | low | p | native | ○ |
| *Paspalum thunbergii* | -0.520 | medium | p | native |  |
| *Luzula capitata* | -0.597 | low | p | native | ○ |
|  |  |  |  |  |  |
| **Species nestedness axis 1** |  |  |  |  |  |
| *Sophora flavescens* | -0.427 | high | p | native | ○ |
| *Polygonatum odoratum* var. *pluriflorum* | -0.450 | medium | p | native |  |
| *Sanguisorba officinalis* | -0.451 | high | p | native | ○ |
| *Liriope minor* | -0.472 | low | p | native | ○ |
| *Potentilla freyniana* | -0.531 | low | p | native | ○ |

**r**: correlation coefficient

**height**: herbaceous plants are classified into three class: low, 0.01~0.3m; medium, 0.3~1m; high, 1~2m

**longevity**: a, annual; b, biennial; p, perennial

**origin**: native or alien

(the above three indices were based on the regional flora (Natural History Museum and Institute, Chiba 2003))

**grassland species**: Species extracted from the list of typical grassland species database in Japan (Koyanagi & Furukawa 2013), or from the list of grassland species defined by a previously-conducted research in the study area (Kaneko, Mimura, Amano, & Hasegawa 2009).
